# Attenuation of Cyclosporine-Induced Sperm Impairment and Embryotoxicity by *Crataegus monogyna* Fruit Aqueous Extract

**Published:** 2013-08-24

**Authors:** Armand Zahra, Najafi Gholamreza, Farah Farokhi, Ali Shalizar Jalali

**Affiliations:** 1 Department of Biology, Faculty of Science, Urmia University, Urmia, Iran; 2 Department of Basic Sciences, Faculty of Veterinary Medicine, Urmia University, Urmia, Iran

**Keywords:** Cyclosporine, Sperm, *In Vitro Fertilization*, *Crataegus monogyna*

## Abstract

**Objective:**

Cyclosporine (Cs), a cyclic undecapeptide with potent immuno suppressive activity, causes several adverse effects including reproductive toxicity. This study aims to examine the ability of *Crataegus monogyna* aqueous fruit extract as an antioxidant to protect against Cs-induced reproductive toxicity.

**Materials and Methods:**

In this experimental study, 32 adult male Wistar rats were divided into four groups of eight animals each. Rats in two groups received 40 mg/kg/day Cs for 45 days by oral gavage. In addition, one of the two groups received *Crataegus monogyna* aqueous extract at a dose of 20 mg/kg/day orally four hours after Cs administration. The remaining two groups consisted of a vehicle treated control (Cont) group and a Crataegus monogyna control (Cr) group. Differences between groups were assessed by analysis of variance (ANOVA) using the SPSS software package for Windows.

**Results:**

Cs treatment caused a signiifcant decrease in sperm count and viability with an increase in DNA damage and protamine deifciency of the sperm cells. We observed signiifcant decreases in fertilization rate and embryonic development, in addition to an increased rate of embryo arrest in Cs-treated rats. *Crataegus monogyna* co-administration attenuated all Cs-induced negative changes in the above-mentioned parameters.

**Conclusion:**

Supplementation with *Crataegus monogyna* a queous fruit extract could be useful against reproductive toxicity during Cs treatment in a rat model

## Introduction

Cyclosporine (Cs), a neutral lipophilic cyclic undecapeptide (C_6_H_11_N_11_O_12_), is an isolate of the Topocladium inlfatum gams extractand a member of the Fungi imperfecti family (1). Since the early 1980s, Cs has been successfully used to prevent organ transplant rejection. It has signiifcantly in-creased graft survival following renal, cardiac, pancreatic, bone marrow and hepatic transplan-tation and decreased patient mortality (2). In ad-dition, Cs is used as a treatment for autoimmune diseases such as idiopathic nephritic syndrome (3), uveitis (4), psoriasis (5) and rheumatoid arthritis (6). The immunosuppressant effects of Cs are as-sociated with its speciifc ability to inhibit signal transductions through the T-cell receptor. This in-hibition results in the prevention of cytokine pro-duction that would normally stimulate an immune response (7). Despite its therapeutic importance, it has been reported that Cs causes renal, hepatic and cardiac damage (8) as well as reproductive toxici-ty in humans and experimental animals (9-13). Although the precise mechanism by which Cs causes reproductive toxicity is still unclear, numerous studies have shown that overproduction of reac-tive oxygen species (ROS) and lipid peroxidation are causative factors involved in Cs-induced ad-verse effects (14-16). Additionally, Cs induces di-rect damage to the hypothalamic-pituitary-gonadal axis (17-19) and Sertoli cell phagocytic function (20) which leads to reproductive toxicity. Hence, a combination of the drug delivery together with potent and safe antioxidant may be the appropriate approach to ameliorate Cs-induced reproductive toxicity. 

Hawthorn (Crataegus spp.), a member of the Rosaceae family, is native to the Mediterranean region, North Africa, Europe and Central Asia and comprises more than 200 species worldwide (21).Hawthorn has been initially indicated as a drug in "Tang-Ben-Cao" (659A.D.), the world’s old-est ofifcially published pharmacopoeia. *Crataegus laevigata* (syn. *Crataegus oxyacantha*) and *Crataegus monogyna* are the two most common species used for medicinal purposes (22). Inde-pendent studies have shown that hawthorn fruit constituents are among the best antioxidants (23-26). Based on this concept, the present study seeks to determine if the aqueous extract of *Crataegus monogyna* fruit with antioxidant properties has a possible protective effect against reproductive tox-icity during Cs treatment in a rat model.

## Materials and Methods

### Plant material

In this experimental study, we collected ripe *Crataegus monogyna* fruits from its natural habitat near the city of Urmia in West Azerbaijan Prov-ince, Northwestern Iran. The Research Laboratory of the Botany Department, Faculty of Science, Ur-mia University scientiifcally conifrmed the identi-ifcation of the collected plants.

### Preparation of the aqueous extract

After collection, the fruits were dried for 7-10 days in the shade at room temperature. The dried fruits were subsequently ground and the powder stored in cloth bags at 5˚C until transfer to the lab-oratory for extraction. The method for preparing dry water-soluble plant powders has been previ-ously described (27). Brielfy, dried plant material (25 g) was mixed in 250 mL of distilled water for 15 minutes at 100˚C, followed by rapid ifltration through a crude cellulose iflter, after which the solution was ifltered through Whatman #1 iflter paper. The resultant ifltrate was freeze-dried and the powder stored at -18˚C in a desiccant until use. The average (w/w) yield was 12.4%.

### Animal model

Adult sexually mature male Wistar albino rats were obtained from the Animal Resources Center of the Science Faculty at Urmia Uni-versity. All animals were housed in a specific pathogen-free environment under standard con-ditions of temperature (22 ± 2˚C), relative hu-midity (50 ± 10%) and light (12 hour light/12 hour dark). A standard pellet diet and fresh drinking water were provided ad libitum. Animals were checked daily for occurrence of any toxic signs of drug delivery. All ethical considerations for the studies on animals were considered careful-ly and the experimental protocol was approved by the Ethics Committee for Research o

### Experimental protocol

After a seven-day acclimation period, we randomly divided the rats into four groups that consisted of eight animals each (n=8) as de-scribed below: control group (Cont), *Crataegus monogyna* group (Cr), Cs group (Cs) and Cs-*Crataegus monogyna* group (CsCr). The two experimental groups (Cs and CsCr) were gavaged with 40 mg/kg/day of Cs (Sandimmune®, Novartis Pharmaceutical Corp., Switzerland)that was dissolved in sesame oil. The controls received an equivalent amount of sesame oil. The Cr group was gavaged with Crataegus mo-nogyna aqueous extract at a dose of 20 mg/kg/day.

The CsCr group also received the same dose of this extract four hours after Cs administration. The treatment period was 45 days. The protocol for this study, including doses and duration of treatment for Cs and *Crataegus monogyna*, was designed ac-cording to previous studies (28-30).

### Sampling

Animals were euthanized by cervical dislo-cation following anesthesia with ketamine (75 mg/kg; IP) 24 hours after the last *Crataegus monogyna* treatment. Following euthanasia, a ventral midline skin incision was made to ac-cess the abdomen and the epididymides were carefully separated from the testicles under 20x magnification with a stereo zoom microscope (Model TL2, Olympus Co., Tokyo, Japan).

### Epididymal sperm characteristics

#### Epididymal sperm count

In order to evaluate the sperm parameters, we placed one caudal epididymis in 1 ml of rat 1-cell embryo culture medium (mR1ECM). The cauda was cut into 2-3 pieces and incubated at 37˚C for 10 minutes in a CO_2_ incubator to allow the sperm to swim out of the epididymal tubules. The epididymal sperm concentration was deter-mined by hemocytometer as follows. We dilut-ed the epididymal sperm to 1:20 in mR1ECM medium then transferred approximately 10 μl of the diluted specimen to each of the count-ing chambers of a hemocytometer, which was placed for 5 minutes in a humid chamber to prevent drying. The cells sedimented during this time and were counted with a light micro-scope at×400. The sperm count was expressed as number of sperm per milliliter (31).

#### Epididymal sperm viability

A 20 µl of sperm suspension was mixed with an equal volume of 0.05% eosin-Y. After incuba-tion for 2 minutes at room temperature, the slides were viewed by bright-ifeld microscope with×400 magniifcation. Dead sperm appeared pink whereas live sperms remained unstained. We counted 200 sperm for each sample then calculated the viability percentages (32).

### Assessment of sperm DNA integrity and chroma-tin quality

#### Staining of spermatozoa with acridine orange 

Acridine orange staining was used to assess the cauda epididymal sperm DNA integrity in the experimental groups. Thick smears were fixed in Carnoy’s fixative that consisted of methanol: acetic acid in a 1:3 ratio for at least 2 hours. The slides were stained for 5 minutes and gently rinsed with deionized water. We evalu-ated 200 sperm under fluorescent microscope; sperm cell heads with normal DNA integrity showed green fluorescence, whereas those with diminished DNA integrity stained orange-red (33).

#### Staining of spermatozoa with aniline blue

The acidic aniline blue (AAB) stain discriminates between lysinerich histones and arginine/cysteine-rich protamines. This technique pro-vides a specific positive reaction with lysine residues in nuclear histones and reveals differences in the basic nuclear protein composition of the sperm. Histonerich nuclei of immature spermatozoa are rich in lysine and consequently absorb the blue stain. Protaminerich nuclei of mature spermatozoa are rich in arginine and cysteine and contain relatively low levels of lysine which leads to a negative reaction to aniline blue. In this study, we stained the airdried fixed smears for 7 minutes with 0.5% aniline blue in phosphate buffered saline. The pH was adjusted to 3.5 by acetic acid. Slides were gently rinsed in distilled water and air dried. The percentage of spermatozoa stained with aniline blue was determined by counting 200 spermatozoa per slide under bright ifeld microscope (34).

### Assisted reproductive technique (ART) procedure

#### Oocyte collection

Each female rat was injected subcutaneously with 30 IU pregnant mare’s serum gonadotropin (PMSG, The Netherlands) 48 hours prior to receiving an intraperitoneal injection of 15 IU human chorionic gonadotropin (hCG, Folligon, TheNetherlands). Rats were euthanized 16 hours after hCG administration. Their oviducts were removed and transferred to a petri dish that containedmR1ECM medium. Using a stereo zoom microscope (Model TL2, Olympus Co., Tokyo, Japan), the Ampullary portion was found and oocytes were dissected out. 

#### Sperm preparation and insemination

Samples from each group were prepared from the sperm suspension as described earlier. Spermatozoa were obtained by swimp and capacitated by incubation at 37˚C and 5% CO_2_ for at least 1 hour. The sperm that had a concentration of 1×10^6^ total sperm/ml were added to a 500 µl fertilization drop of oocytecontaining mR1ECM medium. For each animal we divided a total of 20 oocytes into 10 drops. After six hours of incubation at 37˚C under 5% CO_2_, the cumulus cellfree fertilized oocytes were transferred to fresh drops of mR1ECM medium for embryo culture. We covered all of the medium droplets with mineral oil (35).

#### Assessment of fertilization and embryonic development

Fertilized oocytes were evaluated by appearance of the pronuclei and polar bodies under×200 magnification with an inverted microscope.

After approximately 24 hours following the zygotes culture we assessed the two-cell embryo rate. *in vitro* embryonic development was evaluated at 120 hours under phasecontrast microscopy. Intact, fragmented and/or lysed embryos which did not develop were recorded as "arrested embryos". In the present study, the rate of cell lyses was recorded as follows: Type I: fully lysed, necrotic and/or fragmented embryos. Type II: embryos with partially lysed/fragmented blastomeres. Type III: embryos with some lysed/fragmented blastomeres and/or cytoplasmic vesicles (36).

### Statistical analysis

Results are expressed as mean ± SD. Differences between groups were assessed by analysis of variance (ANOVA) using the SPSS software package for Windows. Statistical signiifcance between groups was determined by Tukey multiple comparison post hoc test p values less than 0.05 were considered to be statistically significant.

## Results

### Epididymal sperm characteristics

As seen in table 1, Cs treatment by itself significantly decreased the mean percentage of sperm concentration and viability compared to the control group. Coadministration of Crataegus monogyna aqueous fruit extract significantly protected the Csinduced negative changes in sperm quantity and viability versus the Cs group.

Administration of Cs alone led to a significant increase in the formation of DNA strand breaks in the epididymal sperm compared to the control group. Cs plus *Crataegus monogyna* aqueous fruit extract treatment markedly suppressed Csinduced DNA damage. The percentage of immature spermin Cstreated rats was higher than those of the controls. Administration of Crataegus monogyna aqueous fruit extract along with Cs improved the chromatin quality of epididymal sperm compared with the Cs-treated group (Table 1).

### Fertilization and embryonic development

Table 2 shows the changes in the fertilization rate and embryonic development in response to various treatments. While Cs administration alone caused significant decrease in the rates of fertilization, two-cell embryos and blastocysts with an increase in percentage of arrested embryoscompared to the control group, Cs plus*Crataegus monogyna* aqueous fruit extract treatment showed a marked amelioration in these parameters (Table 2).

**Table 1:** Effect of cyclosporine (Cs) and Crataegus monogyna aqueous fruit extract on epididymal sperm characteristics


	Control	Cs	Crataegus monogyna	Cs + Crataegus monogyna

**Sperm count (106/ml)**	51.75 ± 3.24	19.50 ± 3.16a	52.62 ± 4.92b	46.12 ± 3.94a,b
**Viability (%)**	87.75 ± 2.60	68.87 ± 3.31a	85.62 ± 5.20b	83.62 ± 4.98b
**Positive for acridine orange stain (%)**	6.50 ± 2.20	28.37 ± 3.92a	5.87 ± 2.64b	11.25 ± 4.02a,b
**Positive for aniline blue stain (%)**	8.75 ± 2.71	19.50 ± 3.20a	9.37 ± 3.33b	15.12 ± 3.22a,b


Values are expressed as mean ± SD (n=8). a; Signiifcant differences as compared with the control group at p < 0.05 and b; Signiifcant differences as compared with the Cs group at p < 0.05.

**Table 2:** Effect of cyclosporine (Cs) and Crataegus monogyna aqueous fruit extract on fertilization and embryonic development


	Control	Cs	Crataegus monogyna	Cs + Crataegus monogyna

**Fertilized oocytes (%)**	96.13 ± 0.45	62.49 ± 8.84a	96.55 ± 0.59b	82.07 ± 4.46a,b
**2-cell embryos (%)**	94.87 ± 0.38	57.88 ± 10.36a	92.17 ± 3.05b	90.59 ± 2.78b
**Blastocysts (%)**	72.66 ± 1.66	24.49 ± 4.01a	67.60 ± 1.06b	70.51 ± 1.60b
**Arrestedembryos (%)**	27.32 ± 1.66	75.49 ± 4.01a	32.39 ± 1.05b	29.48 ± 1.60b
**Arrest type I (%)**	4.74 ± 0.27	54.83 ± 8.03a	6.78 ± 2.32b	4.06 ± 1.11b
**Arrest type II (%)**	5.15 ± 1.37	11.83 ± 2.12a	7.48 ± 1.22b	7.16 ± 0.44b
**Arrest type III (%)**	17.42 ± 2.76	8.82 ± 2.77a	18.11 ± 2.39b	18.24 ± 2.21b


The values are expressed as mean ± SD (n=8). a;Signiifcant differences as compared with the control group at p <0.05 and b ;Signiifcant differences as compared with the Cs group at p <0.05.

## Discussion

Cs is the most frequently used medication for the management of solid organ transplantation and immune diseases because of its potent immunosuppressive effect (37). However, effective clinical use of Cs is limited due to adverse effects such as reproductive toxicity as documented in humans and experimental animals (9-13). Hence, a strategy that diminishes the sideeffects of Cs but preserves its therapeutic efifcacy is necessary. Previous studies have shown that ROS-induced oxidative stress and lipid peroxidations may be involved in the pathophysiology of Cs reproductive toxicity (13, 38).

Spermatozoa are germ cells that have a fundamental role in fertilization. Damaged sperm are one of the factors that cause infertility. Mammalian spermatozoa are particularly vulnerable to excessive ROS-induced oxidative damage because of the molecular anatomy of their plasma membranes (39). The sperm cell membrane contains large quantities of polyunsaturated fatty acids which are involved in regulation of sperm maturation, spermatogenesis, capacitation, acrosome reaction and membrane fusion (40). ROS are highly reactive and can attack the unsaturated bonds of the membrane lipids, leading to lipid peroxidation (41). Peroxidation of sperm lipids demolishes the structure of the lipid matrix in the membranes of spermatozoa, which leads to decreased sperm viability and impaired spermatogenesis (42). In the present study Cs treatment has signiifcantly decreased epididymal sperm count and viability compared to the control group, which conifrmed previous reports that Cs induced spermatozoal damage (11, 17-19, 43).

It has been well documented that oxidative damage to mature sperms during their migration from the seminiferous tubules to the epididymides forms immature sperms as an important factor involved in reproductive disorders (44). It has been shown that oxidative stress affects the integrity of the sperm’s genome by causing single and doublestrand DNA break increase.As shown in the present study, exposure to Cs resulted in a signiifcant increase in spermatozoa DNA strand breaks and in the percentage of immature sperm compared to the control. The results of the current study supported the above mentioned ifndings.

There is increasing evidence that failures in fertilization and embryo development are associated with reduced sperm quality and increased rate of DNA damage in spermatozoa (46-48). Previous studies have shown a correlation between poor blastocyst formation and increased sperm DNA damage (49). Protamine deifciency and increased histone remnants in sperm cells result in an impaired fertilization process and embryo development (50-52).

Damaged spermatozoa are the source of free radicals (53), therefore Cs-induced embryo damage during* in vitro* fertilization could be related to the deleterious effect of ROS-producing damaged spermatozoa during* in vitro* insemination of oocytes. It has been shown that* in vitro* incubation of oocytes with ROS-producing spermatozoa lead to oxidative damage of the oocytes or pronucleate embryos, eventually resulting in impaired embryo development (54, 55).

To date a number of studies have shown the beneifts of anti-oxidants in protecting the reproductive system from the deleterious effects of free radicals generated during Cs exposure. Ellagic acid, a phytochemical with radical scavenging capacity, has recently been shown to ameliorate Cs-induced testicular and spermatozoal damages associated with oxidative stress (56). Lycopene has a potential protective effect against Cs-induced reproductive toxicity due to its highly efifcient anti-oxidant properties (13). According to evidence, supplementation with an antioxidant food such as black grapes is protective against Cs-induced oxidant stress and peroxidation in rat ovary tissues (12).

In the present study, coadministration of *Crataegus monogyna* aqueous fruit extract provided effective protection against sperm impairment and embryo toxicity in Cs-treated rats. This status corroborated the fact that *Crataegus monogyna* wasprotective where free radicals had a crucial role in toxicity. Previous studies have shown that hawthorn extract can effectively lessen the extent of oxidative stress in mouse bone marrow cells and the rat brain due to its strong antioxidant activity (57, 58). Recent reports have also demonstrated that *Crataegus monogyna* fruit aqueous extract can effectively prevent oxidative damage to the reproductive system (59, 60).

## Conclusion

The ifndings of our study have indicated that Cs-induced oxidative stress can lead to sperm impairment and embryotoxicity. Concurrent administration of *Crataegus monogyna* aqueous fruit extract effectively counteracted Cs-related oxidative injury through restoration of an antioxidant defense system.
